# Frequency and determinants of domestic violence against Iranian women during the COVID-19 pandemic: a national cross-sectional survey

**DOI:** 10.1186/s12889-021-11791-9

**Published:** 2021-09-23

**Authors:** Arezoo Yari, Hosein Zahednezhad, Reza Ghanei Gheshlagh, Amanj Kurdi

**Affiliations:** 1 Social Determinants of Health Research Center, Research Institute for Health Development, Kurdistan University of Medical Sciences, Sanandaj, Iran; 2 Department of Psychiatric Nursing and Management, School of Nursing & Midwifery, Shahid Beheshti University of Medical sciences, Tehran, Iran; 3 Spiritual Health Research Center, Research Institute for Health Development, Kurdistan University of MedicalSciences, Sanandaj, Iran; 4 Lecturer in Pharmacoepidemiology and Pharmacy Practice, Strathclyde Institute of Pharmacy and Biomedical Science, University of Strathclyde, Glasgow, UK; 5 Department of Pharmacology, College of Pharmacy, Hawler Medical University, Erbil, Iraq

**Keywords:** Domestic violence, Domestic violence against women, COVID-19

## Abstract

**Introduction:**

Domestic violence (social, legal, and health violence) is the most common type of violence against women. Due to factors such as the current quarantine, this type of violence has increased during the COVID-19 pandemic. The present study aimed to assess the frequency of domestic violence against women and identify the risk factor among Iranian women during the COVID-19 pandemic.

**Methods:**

This online cross-sectional study was conducted on 203 Iranian women during May–June 2020. Data were collected using a domestic violence questionnaire, which measured three forms of violence, including physical, emotional, and sexual violence. A link of the questionnaire was distributed among anonymous subjects through social networking apps, such as WhatsApp and Telegram. The subjects were asked to complete the questionnaire based on their experiences during the COVID-19 pandemic. Data analysis was performed using descriptive statistics and a regression model.

**Results:**

The descriptive results showed that the mean domestic violence against women in all the participants was 34.9 (SD: 17.28). In addition, 26.6% (*n* = 84), 26.1% (*n* = 53), and 21.2% of the subjects (*n* = 43) experienced high levels of physical, emotional, and sexual violence during the COVID-19 pandemic, respectively. The regression model also indicated that lower age, illiteracy/primary education, previous marriage(s), and unwanted/unwise marriage were the significant risk factors for domestic violence against women.

**Conclusion:**

According to the results, domestic violence against women is common among Iranian women during the COVID-19 pandemic. Therefore, strategies are urgently needed to prevent and minimize such domestic violence, and such strategies could be adopted through providing educational opportunities, raising awareness, promoting wanted/wise marriage, and providing social support and rehabilitation opportunities to vulnerable social groups, especially vulnerable women.

**Supplementary Information:**

The online version contains supplementary material available at 10.1186/s12889-021-11791-9.

## Introduction

Domestic violence against women refers to physical or mental violence against women by their partners, and one-third of women have been reported to experience this type of violence [1, 2]. Violence is a behavioral pattern characterized by creating fear, threats, or showing harmful/annoying behavior in order to control a person; such violence could be physical (beating), mental (humiliation), financial (husband not giving money to wife, husband hiding his income from his wife), and sexual (forced sexual relationship or expressing sexual dissatisfaction in a belittling manner) [3, 4] Domestic violence against women is a social, legal, and health concern associated with numerous social, psychological, and financial implications, which violate the rights and dignity of women [5, 6]. Domestic violence also refers to the violence occurring between residences living together; this issue differs from intimate partner violence in which violence involves individuals who may not necessarily live together.

Since 1980s and after the establishment of national and international women’s rights organizations, violence against women has attracted great attention as an important concern. Since the early 1990s, effective laws and resources have been put in place to fight violence against women [7]. Garcia-Moreno (2011) considers violence against women to be the most widespread and embarrassing human rights violation [8]. Domestic violence against women is the most common type of violence against women [1, 2]. Several factors have been identified as the potential causes of marital conflicts and the subsequent domestic violence against women in the United States, including arguments over money, jealousy, sexual problems, alcohol and drug abuse, conflicts over children’s issues, husband’s unemployment, wife’s desire to work outside, and pregnancy [9]. According to the Centers for Disease Control and Prevention (CDC), domestic violence against women in the United States costs about $5.8 billion per year, $4.1 billion of which is spent on the healthcare services provided to the victims, and the remaining is spent on overcoming the harmful effects of partner violence on productivity [10].

Following the outbreak of COVID-19 in the early 2020s, new restrictions such as quarantine, stay-at-home orders, closure of schools and various businesses, and the subsequent financial problems might have exposed women to a higher risk of domestic violence [11]. At the outset of the COVID-19 pandemic, the Iranian government canceled public events and Friday prayers and closed schools, universities, shopping centers, markets, and holy shrines. Since these measures were not implemented continuously, several waves of the pandemic have hit Iran, leading to heavy casualties in an ongoing manner.

Recently, domestic violence against women has been added as another public health concern to the impacts of the COVID-19 pandemic as the opportunistic infection spreads. Violence is assumed to increase as families spend more time together, such as at Christmas or summer vacations [12]. A recent meta-analysis in this regard indicated that the frequency of emotional, physical, and sexual violence against Iranian women before the outbreak of COVID-19 was 59, 45, and 32%, respectively [13]. According to the view that an Iranian woman enters her husband’s house in a white wedding dress, she should also leave with a shroud (the same white dress), under no circumstances should a woman separate from her husband and get a divorce. Consequently, many Iranian women endure violence and do not report the cases to avoid the stigma.

Domestic violence against women has increased during the current quarantine since the victims are forced to stay at home, which makes them even more vulnerable [14]. Domestic violence against women is a hidden issue as women are often reluctant to report the cases for various reasons, such as cultural values, finical problems, fear of losing children, distrust of health officials, and limited knowledge [15]. Due to closure of most businesses during the COVID-19 pandemic, couples tend to spend more time together at home, and marital conflicts and disagreements in this period could increase the risk of domestic violence against women.

The present study aimed to assess the frequency of domestic violence against women and identify the risk factor among Iranian women during the COVID-19 pandemic.

## Methods

### Study design

This online cross-sectional study was conducted on Iranian women during May–June 2020. At the time of data collection, the first wave of the corona had begun and protection measures were being strictly followed and Iran was in a state of lockdown.

### Research population and participants

The inclusion criteria were as follows: married and at least 1 year length of marriage and being literate. In order to compare domestic violence total and dimension scores, all the obtained scores were categorized on a scale of 20 to 100, where ≤25, 25–75, and 75 ≤ indicated low, average, and high scores, respectively.

### Data collection

Data were collected using a demographic questionnaire and the domestic violence questionnaire, which was designed through a literature review. The demographic questionnaire contained data on the age of both spouses, duration of marriage, education level of both spouses, couple’s previous knowledge of each other, number of children, and financial status of the family. The domestic violence questionnaire consisted of 17 items and three dimensions, including physical violence (seven items), emotional violence (six items), and sexual violence (four items). In exploratory factor analysis, the three dimensions could explain 57.19% of the variance of domestic violence. The questionnaire items had 5-point Likert responses (never, 1 to 2 times, 3 to 5 times, 6 to 10 times, and more than 10 times) that indicated the frequency of each item of violence during quarantine. The internal consistency of the physical, emotional, and sexual violence dimensions was respectively estimated at 0.90, 0.89, and 0.76 using Cronbach’s alpha coefficient and 0.82, 0.84, and 0.87 using McDonalds’ Omega, respectively. In addition, an alpha coefficient of 0.92 was calculated for the entire questionnaire (Supplementary Table 1) [16].

The link of the questionnaire was designed with Porsline (equivalent to Google Form) and sent to anonymous subjects through social networking apps, such as WhatsApp and Telegram. The subjects were asked to complete the questionnaire based on their experiences during the COVID-19 quarantine. The inclusion criteria of the study were being married and minimum marriage duration of 1 year.

### Data analysis

In order to calculate the total score of domestic violence against Iranian women during the COVID-19 pandemic, the score of each construct was determined separately, and each construct was allocated a weight based on the number of its items. The weighted mean of total domestic violence was calculated by summing up the scores and dividing the obtained value by the number of the items per construct. In this process, we considered a score range of 20–100, with the scores ≤25, 25–75, and ≥ 75 indicating low, moderate, and high total domestic violence, respectively. Notably, the same definition was adopted for the constructs of total domestic violence as well. Data analysis was performed in SPSS version 23 using descriptive statistics (frequency, percentage, mean, and standard deviation) and a crude and adjusted regression model. In all the statistical analyses, the significance level was set at *P* < 0.05. Given the conditions of the study, a linear regression model was used. Normalization of the total score of domestic violence distribution against Iranian women during the COVID-19 pandemic (normality), the linear correlation between dependent and domestic violence distribution against Iranian women during the COVID-19 pandemic (linearity), and the residuals were equal across the regression line (homoscedasticity), and observations were independent of each other (independency). Moreover, univariate and multivariable linear regression models were employed to explore the determinants of domestic violence against Iranian women during the COVID-19 pandemic. Categorical variables were also incorporated into a multifactorial model using a set of indicators. For instance, age had five categories, from which we selected one age group as the reference and developed four binary variables with the values of zero and one. To estimate the scores of domestic violence against Iranian women during the COVID-19 pandemic, the clustering effect and sampling weights were calculated and applied to the descriptive and analytical statistics using random effects models. At this stage, the independents variables were the factor added to the crude regression model. Notably, variable selection was based on a literature search and statistical significance, so that the variables with the *P*-value of more than 0.05 would be eliminated from the model. Finally, the adjusted regression model was employed with the remaining significant variables that were also based on the literature, and the the regression algorithm used at this stage.

## Results

### Participant characteristics

The sample population of this study included 203 women aged 19–65 years. The mean age of the women and their spouses was 38.59 (SD: 8.77) and 42.68 (SD: 9.52) years, respectively. In terms of education level, 3.4% of the women were illiterate or had primary education, and 76.4% had academic education. In terms of income, only 21.9% of the women had adequate income. In addition, 92.6% of the women had two or fewer children, and 6.19% of the women and 9.9% of their husbands had a previous marriage. Moreover, 21.7% of the women and 19.7% of the spouses had a history of underlying diseases. Finally, 90.5% of the women had wanted marriages, and 49.8% had wise marriages (Table 1). According to the obtained results, 50% of the women who were aged ≤25 years, 40% of the women whose husbands were aged ≤25 years, 57.1% of the women who were illiterate or had primary education, and 42.9% of the women with low income or with two children experienced high levels of domestic violence during the COVID-19 pandemic. Descriptive statistics also suggested that 50% of the women who had a previous marriage, 22.7% of the women who had a history of underlying diseases, 63.2% of the women who had unwanted marriages, and 32.4% of the women who had unwise marriages experienced high levels of domestic violence during the COVID-19 pandemic (Table 1).

**Table 1:** Socio-demographic characteristics of participants based on domestic violence levels

Variable	n (%)	Low domestic violence	Moderate domestic violence	Severe domestic violence
		n (%)	n (%)	n (%)
**Wife Age (Year)**				
< 25	12 (5.9)	0 (0.0)	6 (50.0)	6 (50.0)
25–35	66 (32.5)	10 (15.2)	41 (62.1)	15 (22.7)
35–45	84 (41.4)	24 (28.6)	38 (45.2)	22 (26.2)
45–55	33 (16.3)	7 (21.2)	17 (51.5)	9 (27.3)
> 55	8 (3.9)	2 (25.0)	5 (62.5)	1 (12.5)
**Husband Age (Year)**				
< 25	5 (2.5)	0 (0.0)	3 (60.0)	2 (40.0)
25–35	41 (20.2)	10 (24.4)	21 (51.2)	10 (24.4)
35–45	87 (42.9)	19 (21.8)	49 (56.3)	19 (21.8)
45–55	49 (24.1)	9 (18.4)	25 (51.0)	15 (30.6)
> 55	21 (10.3)	5 (23.8)	9 (42.9)	7 (33.3)
**Wife Education**				
Primary/Elementary	7 (3.4)	0 (0.0)	3 (42.9)	4 (57.1)
High school/ Diploma	41 (20.2)	6 (14.6)	19 (46.3)	16 (39.0)
University	155 (76.4)	37 (23.9)	85 (54.8)	33 (21.3)
**Husband Education**				
Primary/Elementary	12 (5.9)	0 (0.0)	7 (58.3)	5 (41.7)
High school/ Diploma	55 (27.1)	8 (14.5)	29 (52.7)	18 (32.7)
University	136 (67)	35 (25.7)	71 (52.2)	30 (22.1)
**Income status**				
Low	14 (6.9)	1 (7.1)	7 (50.0)	6 (42.9)
Average	130 (64.0)	28 (21.5)	67 (51.5)	35 (26.9)
Sufficient	59 (21.9)	14 (23.7)	33 (55.9)	12 (20.3)
**Number of Children**				
0	51 (25.1)	12 (23.5)	30 (58.8)	9 (17.6)
1	64 (31.5)	12 (18.8)	35 (54.7)	17 (26.6)
2	73 (36.0)	16 (21.9)	33 (45.2)	24 (32.9)
3	15 (7.4)	3 (20.0)	9 (60.0)	3 (20.0)
**Wife Previous Marriage**				
Yes	14 (6.9)	2 (14.3)	5 (35.7)	7 (50.0)
No	189 (93.1)	41 (21.7)	102 (54.0)	46 (24.3)
**Husband Previous Marriage**				
Yes	20 (9.9)	4 (20.0)	9 (45.0)	7 (35.0)
No	183 (90.1)	39 (21.3)	98 (53.6)	46 (25.1)
**Wife History of Diseases**				
Yes	44 (21.7)	8((18.2)	26 (59.1)	10 (22.7)
No	159 (78.3)	35 (22.0)	81 (50.9)	43 (27.0)
**Husband History of Diseases**				
Yes	40 (19.7)	5 (12.5)	23 (57.5)	12 (30.0)
No	163 (80.3)	38 (23.3)	84 (51.5)	41 (25.2)
**Wanted Marriage**				
Yes	184 (90.5)	41 (22.3)	102 (55.4)	41 (22.3)
No	19 (9.4)	2 (10.5)	5 (26.3)	12 (63.2)
**Wise Marriage**				
Yes	101 (49.8)	19 (18.8)	62 (61.4)	20 (19.8)
No	102 (50.2)	24 (23.5)	45 (44.1)	33 (32.4)

### Score of domestic violence

The mean score of domestic violence against the tested women was 34.9 (SD: 17.28) (score range: 20–100). Our findings indicated that 47.5, 32%, and 14.75 of the participants experienced low, moderate, or high domestic violence during the COVID-19 pandemic, respectively. In addition, the mean scores of domestic violence in the dimensions of physical, emotional, and sexual violence were 40.36 (SD: 22.79), 32.56 (SD: 19.01), and 28.99 (SD: 16.57), respectively Moreover, 26.6, 26.1, and 21.2% of the women experienced high levels of physical, emotional, and sexual violence during the COVID-19 pandemic, respectively (Table 2). Physical violence during the COVID-19 pandemic was mostly reported by the women aged ≤25 years, those with high school education or a high-school diploma, and those with two children. In addition, experience of restriction during the COVID-19 pandemic was mostly reported by the women aged 45–55 years, those who were illiterate or had primary education, and those with three children. The highest levels of physical, emotional, and sexual violence were reported by the women who had a previous marriage and those who had an unwanted marriage. Moreover, the highest level of emotional violence was reported by the women who had a history of underlying diseases, and the highest levels of physical and emotional violence were reported by the women who had an unwise marriage (Table 3).

**Table 2:** Domestic violence levels against Iranian women during the COVID-19 pandemic

Level of Domestic Violence	Low Domestic Violence	Average Domestic Violence	High Domestic Violence	Total Score
	n (%)	n (%)	n (%)	Mean ± SD
Total Domestic Violence	43 (21.2)	107 (52.7)	53 (26.1)	34.93 ± 17.28
Emotional	51 (25.1)	98 (48.3)	54 (26.6)	40.36 ± 22.79
Physical	86 (42.4)	64 (61.5)	53 (26.1)	32.56 ± 19.01
Sexual	117 (57.6)	43 (21.2)	43 (21.2)	28.99 ± 16.57

**Table 3:** Socio-demographic characteristics of participants based on three constructs of domestic violence

Variable	N (%)	Physical violence	Emotional violence	Sexual violence	Total violence
Wife Age (Year)					
< 25	12 (5.9)	54.76 ± 27.75	45.55 ± 27.71	32.08 ± 16.30	46.17 ± 23.59
25–35	66 (32.5)	39.95 ± 20.72	31.11 ± 16.85	29.46 ± 18.18	34.36 ± 15.72
35–45	84 (41.4)	40.78 ± 25.33	31.98 ± 19.76	26.30 ± 12.44	34.27 ± 17.81
45–55	33 (16.3)	37.05 ± 18.27	33.13 ± 18.06	33.78 ± 22.00	4.90 ± 16.82
> 55	8 (3.9)	31.42 ± 12.59	28.75 ± 11.53	28.75 ± 13.29	29.85 ± 11.83
Husband Age (Year)					
< 25	5 (2.5)	52.00 ± 28.67	44.66 ± 32.45	29.00 ± 17.46	44.00 ± 26.68
25–35	41 (20.2)	39.16 ± 19.57	30.48 ± 13.38	28.65 ± 17.60	33.62 ± 14.02
35–45	87 (42.9)	38.71 ± 23.34	30.65 ± 18.77	26.66 ± 12.81	33.03 ± 16.92
45–55	49 (24.1)	42.74 ± 22.92	35.64 ± 20.77	30.81 ± 18.21	37.43 ± 18.61
> 55	21 (10.3)	41.22 ± 25.48	34.44 ± 21.06	35.00 ± 22.85	37.36 ± 18.90
Wife Education					
Primary/Elementary	7 (3.4)	43.67 ± 19.56	31.90 ± 13.31	50.71 ± 32.20	41.17 ± 15.06
High school/ Diploma	41 (20.2)	46.41 ± 25.20	41.70 ± 24.71	31.70 ± 16.83	41.29 ± 19.97
University	155 (76.4)	38.61 ± 22.09	30.17 ± 16.75	27.29 ± 14.83	32.97 ± 16.21
Husband Education					
Primary/Elementary	12 (5.9)	47.14 ± 18.41	35.27 ± 18.28	35.00 ± 19.88	40.09 ± 13.85
High school/ Diploma	55 (27.1)	42.90 ± 24.07	36.60 ± 21.81	31.45 ± 19.38	37.98 ± 18.63
University	136 (67)	38.74 ± 22.55	30.68 ± 17.68	27.46 ± 14.85	33.24 ± 16.83
Income status					
Low	14 (6.9)	47.75 ± 21.51	36.66 ± 19.91	35.71 ± 23.36	41.00 ± 16.61
Average	130 (64.0)	40.17 ± 23.64	33.12 ± 19.86	29.80 ± 17.11	35.24 ± 18.23
Sufficient	59 (21.9)	39.03 ± 21.14	30.33 ± 16.83	25.59 ± 12.63	32.80 ± 15.02
Number of Children					
0	51 (25.1)	35.12 ± 17.47	27.84 ± 12.73	28.72 ± 18.35	31.04 ± 14.10
1	64 (31.5)	40.66 ± 22.48	34.89 ± 21.47	28.28 ± 15.96	35.71 ± 17.29
2	73 (36.0)	44.50 ± 25.43	33.97 ± 19.83	29.52 ± 16.44	37.26 ± 18.68
3	15 (7.4)	36.76 ± 24.50	31.77 ± 20.58	30.33 ± 14.81	33.49 ± 19.28
Wife Previous Marriage					
Yes	14 (6.9)	52.85 ± 23.89	42.38 ± 25.63	33.21 ± 19.47	44.53 ± 18.24
No	189 (93.1)	39.44 ± 22.50	31.83 ± 18.31	28.67 ± 16.36	34.22 ± 17.04
Husband Previous Marriage					
Yes	20 (9.9)	43.00 ± 24.58	37.33 ± 22.95	27.50 ± 12.08	37.35 ± 16.83
No	183 (90.1)	40.07 ± 22.64	32.04 ± 18.53	29.15 ± 17.01	34.67 ± 17.35
Wife Previous Disease					
Yes	44 (21.7)	40.12 ± 17.28	33.94 ± 17.93	28.75 ± 16.28	35.27 ± 14.58
No	159 (78.3)	40.43 ± 24.14	32.18 ± 19.34	29.05 ± 16.70	34.84 ± 18.00
Husband Previous Disease					
Yes	40 (19.7)	42.28 ± 23.76	35.50 ± 23.55	31.37 ± 18.67	37.32 ± 19.70
No	163 (80.3)	39.89 ± 22.60	31.84 ± 17.74	28.40 ± 16.03	34.34 ± 16.65
Wanted Marriage					
Yes	184 (90.5)	38.52 ± 21.14	31.25 ± 17.34	28.23 ± 15.76	33.53 ± 16.10
No	19 (9.4)	58.19 ± 30.25	45.26 ± 28.53	36.31 ± 22.22	48.48 ± 22.38
Wise marriage					
Yes	101 (49.8)	37.39 ± 20.37	30.72 ± 17.31	29.35 ± 17.25	33.15 ± 16.46
No	102 (50.2)	43.30 ± 24.71	34.37 ± 20.48	28.62 ± 15.95	36.70 ± 17.96

### Factors associated domestic violence against women

The result of the normality analysis of the residuals, as one of the conditions for linear regression analysis, based on the Kolmogorov-Smirnov test indicated that the total score of violence and the scores of the three dimensions of violence had a normal distribution. Furthermore, the results of the crude and adjusted regression models showed a significant association between age and experiencing domestic violence by the studied women during the COVID-19 pandemic (*P* < 0.05). In other words, lower age was correlated with experiencing higher levels of domestic violence by the women, and the women aged ≤25 years experienced the highest level of domestic violence.

Our findings also indicated a significant correlation between education level and domestic violence against women (*P* < 0.05). In other words, being illiterate and having primary education were observed to be the risk factors for experiencing domestic violence against women. Moreover, the crude regression model showed significant correlations between income and the number of children with domestic violence against women, while these associations were not considered significant in the adjusted regression model (*P* > 0.05) (Table 4).

**Table 4:** Association between domestic violence and independent variables in the crude and adjusted models

Variable	n (%)	Average score of total domestic Violence	Crude difference OR (95% CI)	***P***- value	Adjusted Difference (95% CI)	*P*- value
**Wife Age (Year)**						
< 25	12 (5.9)	46.17	16.32 (1.12–31.51)	0.035	22.84 (3.00–18.97)	0.024
25–35	66 (32.5)	34.36	4.51 (−7.94–16.97)	0.478	12.54 (−4.94–13-03)	0.160
35–45	84 (41.4)	34.27	4.41 (−7.89–16.73)	0.482	7.40 (−9.4–12.46)	0.388
45–55	33 (16.3)	4.90	5.04 (−8.07–18.16)	0.451	5.12 (−10.65–17.87)	0.525
> 55	8 (3.9)	29.85	–	–		
**Husband Age (Year)**						
< 25	5 (2.5)	44.00	6.63 (− 10.01–23.27)	0.435	–	–
25–35	41 (20.2)	33.62	−3.37 (−12.71–5.23)	0.414	–	–
35–45	87 (42.9)	33.03	−4.31 (−12.46–3.80)	0.297	–	–
45–55	49 (24.1)	37.43	0.064 (−8.66–8.78)	0.989	–	–
> 55	21 (10.3)	37.36	–	–	–	–
**Wife Education**						
Primary/Elementary	7 (3.4)	41.17	8.25 (4.57–20.98)	0.005	2.95 (7.56–21.03)	0.023
High school/ Diploma	41 (20.2)	41.10	8.32 (2.51–14.12)	0.208	3.12 (0.437–2.108)	0.348
University	155 (76.4)	32.97	–	–	–	–
**Husband Education**						
Primary/Elementary	12 (5.9)	40.09	5.13 (−3.21–16.92)	0.182	–	–
High school/ Diploma	55 (27.1)	37.98	2.72 (0.60–10.09)	0.082	–	–
University	136 (67)	33.24	–	–	–	–
**Income status**						
Low	14 (6.9)	41.00	8.20 (1.77–18.18)	0.017	2.80 (−7.98–12.80)	0.582
Average	130 (64.0)	35.24	2.44 (2.82–7.71)	0.036	1.73 (− 347–6.95)	0.514
Sufficient	59 (21.9)	32.80	–	–	–	–
**Number of Children**						
0	51 (25.1)	31.04	−2.44 (−12.26–7.38)	0.051	–	–
1	64 (31.5)	35.71	2.22 (−7.36–11.82)	0.063	–	–
2	73 (36.0)	37.26	3.77 (−5.71–13.25)	0.069	–	–
3	15 (7.4)	33.49	–	–	–	–
**Wife Previous Marriage**						
Yes	14 (6.9)	44.53	–	–	–	–
No	189 (93.1)	34.22	−10.31	0.029	−10.20 (1.53–12.32)	0.048
**Husband Previous Marriage**						
Yes	20 (9.9)	37.35	–	–	–	–
No	183 (90.1)	34.67	−2.68	0.508	–	–
**Wife Previous Disease**						
Yes	44 (21.7)	35.27	–	–	–	–
No	159 (78.3)	34.84	−4.25	0.885	–	–
**Husband Previous Disease**						
Yes	40 (19.7)	37.32	–	–	–	
No	163 (80.3)	34.34	−2.97	0.327	–	–
**Wanted marriage**						
Yes	184 (90.5)	33.53	–	–	–	–
No	19 (9.4)	48.48	14.94 (7.06–22.82)	0.000	10.10 (1.33–18.88)	0.024
**Wise marriage**						
Yes	101 (49.8)	33.15	–	–	–	–
No	102 (50.2)	36.70	3.55 (1.16–9.26)	0.014	1.67 (7.19–25.68)	0.032

* X/df index =2.15, The Goodness-of-fit Index (GFI) =0.934, RMSEA index = 0.069, AGFI index = 0.941

According to the results of the crude and adjusted regression models, the wife’s previous marriage was significantly correlated with domestic violence (*P* < 0.05) as women without a previous marriage reported experiencing lower levels of domestic violence during the pandemic. However, the husband’s previous marriage was not significantly correlated with domestic violence during the COVID-19 pandemic. The previous marriage of women and their husbands was not significantly correlated with domestic violence against women (*P* > 0.05). The results of the raw and adjusted regression models also showed a significant association between wanted and wise marriage with domestic violence against women during the pandemic (*P* < 0.05) as the women with an unwanted or unwise marriage reported experiencing higher levels of domestic violence during the COVID-19 pandemic (Table 4).

## Discussion

The present study aimed to assess the frequency of domestic violence against women and identify its determinants among married Iranian women during the COVID-19 pandemic. According to the obtained results, the majority of the participants (52.7%) experienced moderate levels of domestic violence. In a meta-analysis conducted by Hajnasiri et al. (2016), the prevalence of domestic violence against women in Iran was estimated at 66% [1]. In a study by Nouri et al. (2006), which aimed to evaluate the status of domestic violence against women in Kurdistan province (Iran), the obtained results indicated that 79.7, 60, and 32.9% of women experienced mental, physical, and sexual violence, respectively [17]. Previous studies have also indicated that domestic violence against women is correlated with teenage pregnancy, unwanted pregnancy, sexually transmitted infections, malnutrition, and adverse birth outcomes [18–21]. Furthermore, exposure to domestic violence could make women vulnerable to depression and alcohol or drug abuse by reducing their self-esteem [19, 20, 22].

In the present study, domestic violence against women was most frequently reported by the women aged ≤25 years, those with lower education levels, those with a previous marriage, and those with an unwanted marriage. This finding is consistent with the risk factors for domestic violence against women that have been reported by CDC, including young age, poverty, economic stress, and low education levels [23]. In line with our findings, Gerino et al. reported several risk factors for domestic violence against women, including lower age, physical problems, unemployment, financial problems, and life stress [24]. Previous findings have shown that education level is negatively correlated with domestic violence against women, and higher education is considered to be a protective factor against domestic violence, which reduces the risk of domestic violence against women, especially sexual violence [17, 25]. Several studies have highlighted the role of age as a protective factor of domestic violence, and younger women are reported to be more vulnerable to domestic violence [26, 27], which is in line with the results of the present study. It seems that with age, couples tend to better understand each other and become more able to solve their marital problems rationally.

In the current research, a positive correlation was observed between domestic violence against women and low income, which is consistent with the previous findings in this regard [28–30]. This finding could be explained by the fact that with the spread of COVID-19 and the subsequent quarantine, families experienced more economic problems due to recession or loss of jobs; the negative impact of these financial problems is felt by low-income families more intensely. On the other hand, our findings indicated that the women with lower education levels experienced more domestic violence. In line with this finding, previous studies have demonstrated that women with lower education levels often experience more psychological problems due to domestic violence compared to those with higher education [31, 32]. A study conducted on Indian and Bangladeshi women showed that higher education is a protective factor against domestic violence against women [33]. Women with higher education are more capable of controlling and managing potentially violent situations compared to those with lower education.

According to the results of the present study, wanted and wise marriages were significantly correlated with domestic violence against women during the COVID-19 pandemic as women with a wanted/wise marriage reported experiencing lower levels of domestic violence during the pandemic. Similarly, Yari et al. stated that forced marriage was significantly correlated with intimate partner sexual violence [25]. The risk factors for domestic violence against women observed in the present study are consistent with the previous findings in this regard. Notably, the spread of COVID-19 infection has deteriorated this situation since numerous women do not have access to support services and are vulnerable to higher levels of stress due to domestic violence against women.

With the spread of the COVID-19 infection and the subsequent quarantine, people are forced to spend more time at home, which could have increased the risk of domestic violence against women due to possible disagreements, fear of contracting the virus, and financial problems. In addition, children stay at home longer due to the closure of schools, which could also be a significant source of stress for mothers [34]. In France, a 32% increase was reported in domestic violence against women in the first week of quarantine. Furthermore, the first 2 weeks of quarantine was accompanied by a 60, 18, and 250% increase in domestic violence against women in Argentina, Brazil, and Colombia, respectively. During the quarantine in Australia, internet searches to seek help by domestic violence victims increased by 75% [35].

Evidence suggests that increased domestic violence against women is a global concern that is not limited to certain countries. During the pandemic, numerous victims of domestic violence may be unable to report the cases due to being stuck at home [36]. Given the increased risk of domestic violence against women during the COVID-19 pandemic, proper policies should be adopted to support women during quarantine [37]. In addition, our experiences during the current pandemic could help us better manage future crises. The victims of domestic violence against women should have access to consultation, shelter, and 24-h hotlines. One of the limitations of this study was the small sample size. Because the distributed questionnaire link was only active for 1 month, our sampling was done at the same time, which caused us to take a small sample. Some women also feared that their husbands would find out that they had participated in the study, causing them to just open the link and read the questions but not complete them.

## Conclusion

According to the results, the increased risk of violence against women during epidemics, including the COVID-19 pandemic, emphasizes the importance of paying attention to the underlying social factors contributing to this issue. In this regard, we recommended some measures to prevent domestic violence against women, such as providing women and other vulnerable social groups with educational opportunities, raising the awareness of young people, promoting wanted and wise marriages, and providing social support and rehabilitation opportunities to vulnerable women (e.g., women with disabilities or those with previous marriages). Our battle against COVID-19 should encompass strategies to provide women (especially vulnerable women) with social and healthcare support.

## Supplementary Information

**Additional file 1: Supplementary Table 1.** The items of domestic violence during quarantine scale.

## Data Availability

The datasets used and/or analysed during the current study are available from the corresponding author on reasonable request.
