# Supplementary material for: Frequency and determinants of domestic violence against Iranian women during the COVID-19 pandemic: a national cross-sectional survey
Source: BMC Public Health. 2021 Sep 23;21:1727. doi: 10.1186/s12889-021-11791-9 (PMC8457898; doi:10.1186/s12889-021-11791-9)
Supplement: Supplementary file 1 — Additional file 1: Supplementary Table 1. The items of domestic violence during quarantine scale. [file 12889_2021_11791_MOESM1_ESM.docx]

Supplementary Table 1: The items of domestic violence during quarantine scale

| No | Items | Never | 1 to 2 times | 3 to 5 times | 6 to 10 times | more than 10 times |
| --- | --- | --- | --- | --- | --- | --- |
| 1 | He has accused you of laziness, indifference, or not performing your duties towards him or household chores. |  |  |  |  |  |
| 2 | He has underestimated your ability to raise children and accused you of not being a successful mother/wife. |  |  |  |  |  |
| 3 | He has blamed you by belittling your thoughts and interests. |  |  |  |  |  |
| 4 | He has not shown affection towards you, causing you to feel severe loneliness. |  |  |  |  |  |
| 5 | He has belittled your clothing preferences, body, or appearance. |  |  |  |  |  |
| 6 | He has insulted your loved ones. |  |  |  |  |  |
| 7 | He has ignored you intentionally or refrained from having sex with you for weeks. |  |  |  |  |  |
| 8 | He has threatened you with physical harm. |  |  |  |  |  |
| 9 | He has beaten you up (slapping, kicking, pulling hair) |  |  |  |  |  |
| 10 | He has threatened you with divorce. |  |  |  |  |  |
| 11 | He has thrown objects at you. |  |  |  |  |  |
| 12 | He has insulted you in front of others. |  |  |  |  |  |
| 13 | He has been away for days/weeks without informing you. |  |  |  |  |  |
| 14 | He has not allowed you to eat or choose your food. |  |  |  |  |  |
| 15 | He has not allowed you to use telephone or social media. |  |  |  |  |  |
| 16 | He has not allowed you to choose your favorite TV channel. |  |  |  |  |  |
| 17 | He has forced you to have sexual intercourse when you were not interested. |  |  |  |  |  |
